# Plant Ontogeny, Spatial Distance, and Soil Type Influence Patterns of Relatedness in a Common Amazonian Tree

**DOI:** 10.1371/journal.pone.0062639

**Published:** 2013-05-07

**Authors:** Carlos Eduardo A. Barbosa, Tracy M. Misiewicz, Paul V. A. Fine, Flávia R. C. Costa

**Affiliations:** 1 Graduate Program in Ecology, Instituto Nacional de Pesquisas da Amazônia (INPA), Manaus, Amazonas, Brazil; 2 Department of Integrative Biology, University of California, Berkeley, California, United States of America; 3 Coordenação de Pesquisas em Biodiversidade, Instituto Nacional de Pesquisas da Amazônia (INPA), Manaus, Amazonas, Brazil; USDA-ARS, United States of America

## Abstract

The formation of spatial genetic structure (SGS) may originate from different patterns of seed deposition in the landscape, and is mostly determined by seed dispersal limitation. After dispersal, mechanisms such as filtering by environmental factors or attack by herbivores/pathogens throughout plant development stages, and potentially either disrupt or intensify SGS patterns. We investigated how the genotype of *Protium subserratum* (Burseraceae), a common tree species in the Ducke Reserve, Brazil, is distributed across the landscape. We used seven microsatellite markers to assess the SGS among plants at different life stages and in different environments. By quantifying the patterns of relatedness among plants of different sizes, we inferred the ontogenetic stage in which SGS changes occurred, and compared these effects across soil types. Relatedness among seedlings decreased when distance between seedlings increased, especially for the youngest seedlings. However, this trend was not continued by older plants, as relatedness values were higher among neighboring individuals of the juvenile and adult size class. Contrasting relatedness patterns between seedlings and larger individuals suggests a trade-off between the negative effects of being near closely-related adults (e.g. due to herbivore and pathogen attack) and the advantage of being in a site favorable to establishment. We also found that soil texture strongly influenced density-dependence patterns, as young seedlings in clay soils were more related to each other than were seedlings in bottomland sandy soils, suggesting that the mechanisms that create and maintain patterns of SGS within a population may interact with environmental heterogeneity.

## Introduction

Patterns of positive spatial genetic structure (SGS) derive from dispersal limitation which results in closely-related seeds of a given plant species being deposited close to one another [1], [2], with genotypes being spatially distributed in a non-random fashion [3]. However, once a seed arrives at a site, both abiotic and biotic factors affect plant recruitment, which ultimately determines the distribution of individuals in the landscape [4], and thus SGS patterns become rearranged over time. Microhabitat characteristics can be particularly important in the adjustment of SGS as seedling development proceeds, because factors that promote seedling success or mortality such as pathogens, predation, competition and resource availability can differ drastically at very small scales [5]–[7].

In addition to each species’ dispersal capabilities, the temporal asynchrony of seed production and fecundity variation among adults in a population also influence population-level patterns of SGS. For example, if each reproductive event in a population represented a different set of individuals producing seeds within a given year, greater positive SGS would be found within individuals of that year’s offspring, and the relatedness of seedlings among different plant size classes would be reduced. Also, adults with higher fecundity, which disproportionally contribute to the seed shadow composition [8], should produce larger numbers of seedlings, leading to two possible outcomes. One is the increase in the chance that more individuals of the descendents of these “superfecund” adults would reach adulthood. In this scenario adults would be more related to each other than would be expected by chance. Furthermore, greater production of seeds would enhance the probability of more distant seed dispersal, which could influence the SGS within less fecund offspring, by the input of seeds with different genotypes. On the other hand, if most mother trees in a population produce seeds every year, this would mean that the majority of seeds in any given location should be closely-related. In this case, the pattern of positive SGS might prevail despite the additional input of seeds with different genotypes dispersed from adults with asynchronous reproduction.

Patterns of SGS that emerge in the seed shadow of the maternal tree may also be influenced by density-dependent or distance-dependent mortality. The establishment success of propagules should be inversely related to the distance from their maternal parent, because of the higher probability of attack by specialist herbivores and pathogens that are associated with maternal trees [9], [10]. This mechanism is the core of the Janzen and Connell hypothesis, which predicts that density and/or distance-responsive natural pathogens and herbivores cause a disproportionate amount of mortality near adult trees [9]–[11]. This hypothesis could be extended to the intraspecific level, especially in cases when variation in resistance to specialist herbivores or pathogens is present in a plant population, and considering that mother trees and their seedlings are more likely to share phenotypes involved with resistance to enemies [12], [13]. In this way, the natural enemies associated with the mother tree that attack neighboring seedlings could be targeting genotypes more similar to the adult, which would result in disruption of the SGS patterns predicted by seed dispersal limitation. Thus the positive patterns of SGS in individuals from the youngest seedling stages would be predicted to disappear in older development stages. Furthermore, the degree of positive SGS disruption should thus be regulated by the strength of the density/distance-related mortality.

Although the proximity to conspecific adults might lead to increased density or distance-related seedling mortality, for a shade-tolerant tree species the site where a reproductive conspecific adult has successfully established also may be most likely to offer “optimal” abiotic conditions for seedling establishment [14], [15]. In this scenario, there would be a tendency of seedlings to recruit to adulthood around their maternal tree, with positive SGS being detectable throughout all development stages of the species, especially in stages older than the youngest seedlings if the advantage in being in an optimal site increased with developmental stage.

In addition, the periodicity of fruit production, the amount of fruit produced and/or dispersed, density and distance-dependent mortality and even the suitability of a site for seedling recruitment is also likely to interact with environmental heterogeneity. Resource availability influences both the amount of fruit produced [16], [17] and the periodicity of the reproduction events of a tree population [8]. Additionally, seedling establishment may be affected by highly variable abiotic (water, nutrients and light availability [18]) or biotic factors, including litter depth [19], competition [5] and natural enemies [20]), that might result in “optimal” sites being more common at particular habitats across an environmental gradient. Finally, plant mortality due to herbivore attacks may differ according to soil characteristics [21]. Given the variation of seedling establishment with environmental factors, SGS patterns might vary within a population that spans an environmental gradient across different habitat types.

The processes involved in the formation of SGS patterns described above are not mutually exclusive and could occur simultaneously and/or have stronger effects at different age classes. Here, we quantify how the relatedness patterns among individuals of a common Amazonian tree species change with ontogenetic stage from very young seedlings to adults. Second, we investigate the effect of habitat characteristics (represented here by soil texture) in driving SGS patterns.

## Methods

### Study Species and Study Site

*Protium subserratum* (Engl.) Engl. (Burseraceae) is a widespread species found in northern Amazonia, extending into Guyana, French Guyana, the Brazilian Amazon, Ecuador, Peru, Colombia and Venezuela. This dioecious tree species occurs across a range of soil types including clay, sandy clay and white sand [22] and its red fruits are thought to be dispersed by birds and monkeys [23]. We studied a population of *P. subserratum* along an edaphic and topographic gradient within the Reserva Florestal Adolpho Ducke (hereafter referred as Ducke), where this species is found across a soil gradient that transitions from sandy soil in low elevation areas (39 m a.s.l.) to more nutrient rich clay soil at higher elevation areas (109 m a.s.l.).

**Figure 1:**
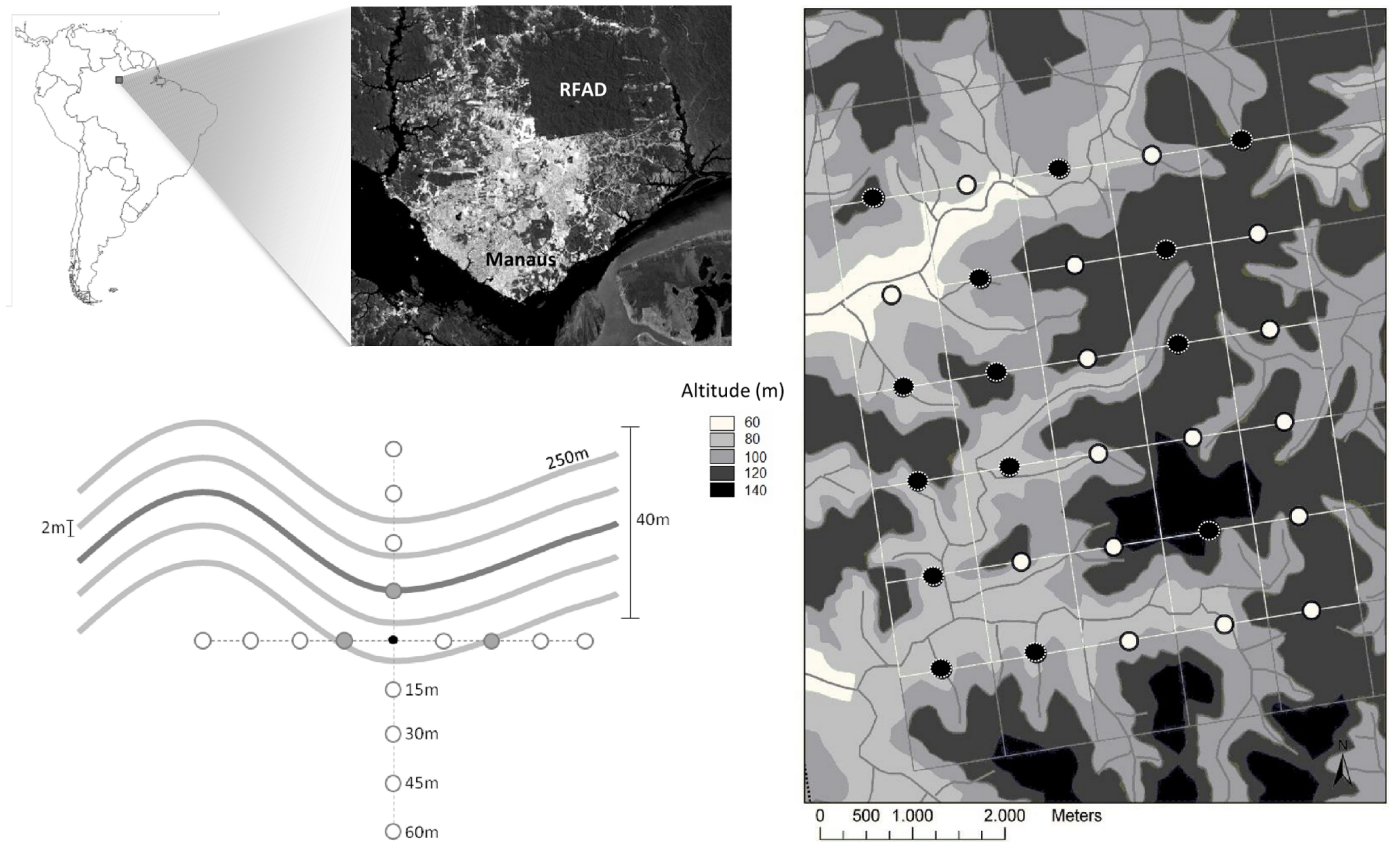
Site location and sampling design. Study site location in the Central Amazon, north of Manaus, Brazil. The trail grid is shown in the right figure and the plots that contained sufficient individuals for SGS analyses are represented by black dots. Topographical variation is shown in a grey-scale, from low (white) to high areas (black). In the lower left of the figure the sampling scheme is depicted, including 5 sampling lines of 2 m width and 250 m length. The extra sampling area conducted around each adult (black dot), is represented by points around it (open circles), excluding areas previously sampled in the lines (gray circles).

Ducke is a *terra firme* tropical forest that covers an area of 100 km^2^ at the northern limit of the city of Manaus (02°55′S, 59°59′W, Fig. 1) in the Brazilian state of Amazonas. It is a closed canopy forest, with trees reaching 35–40 m height [24] and includes a high abundance of understorey palms [25]. Ducke is under the jurisdiction of the Brazilian National Institute for Amazon Research (INPA), which issued permits for the sampling involved in the present study. The studied species is not an endangered or protected species.

### Study Design

We conducted our study in 30 of the 72 permanent plots that were previously set up as part of the Brazilian Long-Term Ecological Research Program and the Research Program in Biodiversity [26]. The plots are systematically distributed within a trail grid system, 1 km distant from each other, and are 250 meters long with the width of each transect varying according to the taxa or the life stage to be studied [27]. Tree size classes define the plot width, which is 4 m for DBH ≥1 cm, 20 m for DBH ≥10 cm and 40 m for DBH ≥30 cm. Within these limits all trees are mapped and tagged. The plots follow the altitudinal contours, which results in a constant elevation throughout the plot length, important at Ducke, where topography is highly correlated with clay content [28].

### Data Collection

In each of the 30 plots the sampling took place along the 250 meter transect, in four lines (2 m wide) that were parallel to the central line (Fig. 1). We counted and marked the geographic location of all *P. subserratum* seedlings and saplings along these lines with a handheld GPS unit and estimated the height of each individual. Leaflets were collected and dried in silica-gel for genetic analysis. Juveniles and adults that were outside the collection lines but were within the plots and were mapped from previous studies were also sampled. To expand the sample of each adult’s seed shadow extra seedlings were sampled around adults in a cross-shaped sampling design in concentric circles around the trunk (Fig. 1).

Sampled individuals were categorized in four size classes: seedlings up to 20 cm in height (first-year seedlings), seedlings 21 to 40 cm in height (young seedlings), seedlings from 41 and 100 cm in height (saplings) and all juvenile and adult trees taller than 200 cm (juveniles and adults) (Table 1). Only four individuals in the 100–200 cm size class were counted in all of the plots and as a result this size class was excluded from our study. Because all subsequent analyses rely on the comparison of pairs of individuals, only plots that had at least two individuals in any size class were included (14 of the 30 plots).

**Table 1:** Number of individuals from the four different size classes at each plot, soil category (sandy for less than 15% of clay in soil composition, and clay for more than 15% of clay) and the total number of seedlings included in the genetic analysis for each size class.

Plot	Soil type	Clay %	First-year seedlings	Young seedlings	Saplings	Juveniles& Adults	Total
lo8_1500	sandy	2.68	11	14	3	3	**31**
lo8_0500	sandy	2.74	16	8	4	1	**29**
lo4_1500	sandy	3.79	6	5	1	1	**13**
lo3_2500	sandy	4.99	10	11	3	3	**27**
lo6_1500	sandy	11.81	1	1	3	1	**6**
lo7_0500	sandy	13.04	7	5	7	1	**20**
lo6_0500	clay	32.24	5	11	3	1	**20**
lo5_2500	clay	42.57	12	11	6	1	**30**
lo3_0500	clay	62.40	9	14	10	5	**38**
lo7_3500	clay	72.46	22	10	5	5	**42**
lo4_3500	clay	77.33	25	12	2	4	**43**
lo3_4500	clay	78.28	5	6	4	1	**16**
lo5_1500	clay	81.31	7	12	3	4	**26**
lo5_3500	clay	83.30	6	10	3	0	**19**
**Total**			**142**	**130**	**57**	**31**	**360**

Information on soil texture for each plot was gathered from previously collected data that is available at http://ppbio.inpa.gov.br/knb/metacat/fecosta.4.5/ppbio. At Ducke soil texture strongly influences plant community composition [29]. Schietti et al. [30] have shown that despite the fact that soil texture changes in a continuous fashion, plant composition changes abruptly when soils shift from being very sandy (less than 15% of clay in its composition) to being higher in clay content. Accordingly, we divided the soils into two classes: “sandy” plots when they contained less than 15% clay and “clay” plots when clay content was greater than 15%.

### Genetic Data

#### Microsatellite genotyping and genetic diversity

Genomic DNA was extracted from 360 individuals (Table 1) using a DNEasy Plant Mini Kit (Qiagen, Santa Clarita, California, USA) and all samples were genotyped using seven microsatellite markers specifically developed for *P. subserratum* (Prot28, Prot29, Prot67, Prot70, Prot71, Prot78, Prot83 - [31]). Polymerase chain reactions (PCR) were performed in a total reaction volume of 12.30 µL containing 1 µL genomic DNA, 6.30 µL 2X GoTaq Green Master Mix (Promega, Madison, Wisconsin, USA), 0.4 µM of un-tagged primer, 0.2 µM of tag-modified primer and 0.2 µM of M13R primer fluorescently labeled with 6-FAM or Hex [32] and DNase free water. Amplifications were initialized with a denaturation step of 2 min and 30 s at 95°C, followed by four touch-down cycles, repeated five times each, denaturation at 95°C for 20 s, annealing temperatures of 55, 53, 50, 49°C, respectively for each touch-down step, for 20 s, followed by a ramp of 2°C/s to 45°C, extension temperature at 72°C for 30 s, and a final cycle of 95°C for 20 s, 45°C for 20 s, 72°C for 30 s, repeated 15 times with a final extension at 72°C for 10 minutes. The allele sizes of the resulting fluorescent labeled fragments were determined using the size standard LIZ-500 on an ABI 3730 DNA Analyzer (Applied Biosystems, Foster City, California, USA). Peaks were scored using Peak Scanner version 1.0 (Applied Biosystems).

Number of alleles (A), observed (H_o_) and expected heterozygosity (H_e_) were calculated in GenAlEx version 6.1 [33]. Departure from Hardy-Weinberg equilibrium and linkage disequilibrium were calculated using Genepop 4.0.10 [34] and probability of null alleles was calculated with Cervus 3.0 [35].

### Spatial Distribution of Genetic Variation across the Landscape

The distribution pattern of genetic variation across the landscape was assessed using spatial autocorrelation analysis, to examine genetic relatedness as a function of the spatial separation among individuals. This multivariate approach was used to test the autocorrelation (*r*) between geographic distance (expressed in meters between each pair) and genetic distance (a Euclidean distance measure) matrices [36] at the plot scale (250 m). The coefficient *r* ranges from –1 to 1 and gives a measure of genetic similarity between individuals within each distance class (positive values refer to degree of relatedness and negative values mean individuals are less related than expected at random). It is similar to Moran’s – *I^(h)^* coefficient, except that *r* accounts for the number of times an individual is paired with other individuals in the same distance class, while Moran’s *I* considers an individual pairing at any distance class in a binary way (paired/not paired). In order to determine if null alleles could be influencing the *r*-value calculations, additional estimates of relatedness were calculated using ML-Relate [37], which accommodates the presence of null alleles, to determine the effect of null alleles relative to our initial relatedness estimates.

The null hypothesis of random distribution of genotypes was created by randomly permuting the genotypes among the sampling locations. After repeating the permutation 999 times it was possible to calculate *r*-values and then create an empirical null distribution for each distance class, allowing the construction of a confidence interval (95% CI). Significant autocorrelation was inferred each time an observed *r*-value occurred outside the confidence interval. The error bars calculated for each *r*-value also give information on the significance of the autocorrelation, in which significant autocorrelation at each distance class was inferred only when the error bar did not cross the x-axis. In order to determine if the observed autocorrelation values represent credible evidence of fine scale genetic structure and to test all the distance classes, *p*-values were calculated using the methods of Smouse et al. [38]. The *p*-values were then used to calculate a probability metric *ω* [39] following Smouse et al. [38], which allowed us to evaluate the hypothesis of no autocorrelation at any distance.

To evaluate the extent of the seed shadow overlap on patterns of SGS in each life stage and the effect on relatedness patterns among plants at different life stages, we performed a “between-generation” analysis [40], in which relatedness is calculated for pairs of individuals including a seedling (first-year seedling, young seedling or sapling) and an adult, yielding the degree of relatedness of each seedling with their closest adults. A “within-generation” analysis was also undertaken to measure the relatedness among seedlings of each size class with all individuals pooled together. A similar within and between generation relatedness is expected when the seed shadow overlap is low, because very young seedlings have greater probability of being most closely related to their closest adult. Higher relatedness within seedlings would indicate that they share parents outside the sampling area, and that those adults contribute substantially to the plot seed rain composition. The relatedness index used was Nason’s kinship coefficient *Fij* [41], calculated on SPAGeDi 1.3 [42]. The coefficient of relatedness (*r*) used is the double of the value of *Fij* assuming neither individual is inbred [43].

In order to explore the effect of developmental stage and environmental features on the spatial organization of individuals, autocorrelation tests were performed independently with all samples pooled and also according to plant size class and soil type. In the analyses comparing spatial patterns between soil types, all seedlings up to 40 cm were pooled to increase sample sizes, so that at least one pair of individuals was compared in each distance class; fewer than two individuals in some distance classes precluded analysis of soil-related patterns for larger size classes. To evaluate if the differences among the fine-scale genetic structure patterns of the size and soil types were significant, we used a non-parametric heterogeneity test. This encompasses the autocorrelation coefficient values calculated for each lag of each group and the pooled correlogram as the null hypothesis reference frame. This test accounts for differences in sample size by applying a weighted average bias correction [38]. All correlograms and related tests were undertaken in GenAlEx v.6.1 [33]. To minimize the effects of incomplete genotypes the interpolation option was applied when making the genetic distance matrices.

## Results

### Genetic Diversity

The number of alleles per locus ranged from four (Prot71 and Prot78) to 12 (Prot29), with a mean value of 7.71 alleles per locus. Mean expected heterozygosity (H_e_) was 0.66 and observed heterozygosity (H_o_) was 0.60 (Table 2). Of the seven microsatellites, only one (Prot 71) departed from Hardy-Weinberg expectations. Null alleles were detected in loci Prot67 (*P* = 0.071), Prot70 (*P* = 0.068) and Prot 78 (*P* = 0.068) however, correcting for the presence of null alleles and recalculating the *r*-values using ML-Relate resulted in no significant change, corroborating our initial *r*-values. Accordingly, our initial *r*-values were used in all further analyses.

**Table 2:** Summary of genetic diversity. Sample size (*n*), average allele number per locus (Na), effective allele number (Ne), number of private alleles and frequencies in parenthesis (Pa), observed heterozygosity (H_o_), expected heterozygosity (H_e_) and fixation index (F). Standard error values are within parenthesis.

Groupings	*n*	Na	Ne	Pa	H_o_	H_e_	F
First-year sdls.	142	6.4(0.99)	3.3(0.43)	3(0.004)	0.6(0.04)	0.7(0.04)	0.1(0.04)
Young sdls.	130	6.7(0.84)	3.3(0.42)	3(0.011)	0.6(0.04)	0.7(0.04)	0.1(0.02)
Sapling sdls.	57	6.4(0.90)	3.1(0.43)	1(0.01)	0.6(0.04)	0.6(0.04)	0.0(0.05)
Adult/juvenile	30	5.3(0.75)	2.8(0.26)	1(0.017)	0.6(0.07)	0.6(0.04)	0.0(0.07)
Clay	166	6.7(0.89)	3.2(0.47)	7(0.010)	0.6(0.04)	0.6(0.04)	0.0(0.01)
Sand	74	6.1(0.67)	3.2(0.36)	3(0.028)	0.5(0.04)	0.7(0.04)	0.2(0.06)

### Spatial Genetic Structure and Plant Size

**Figure 2:**
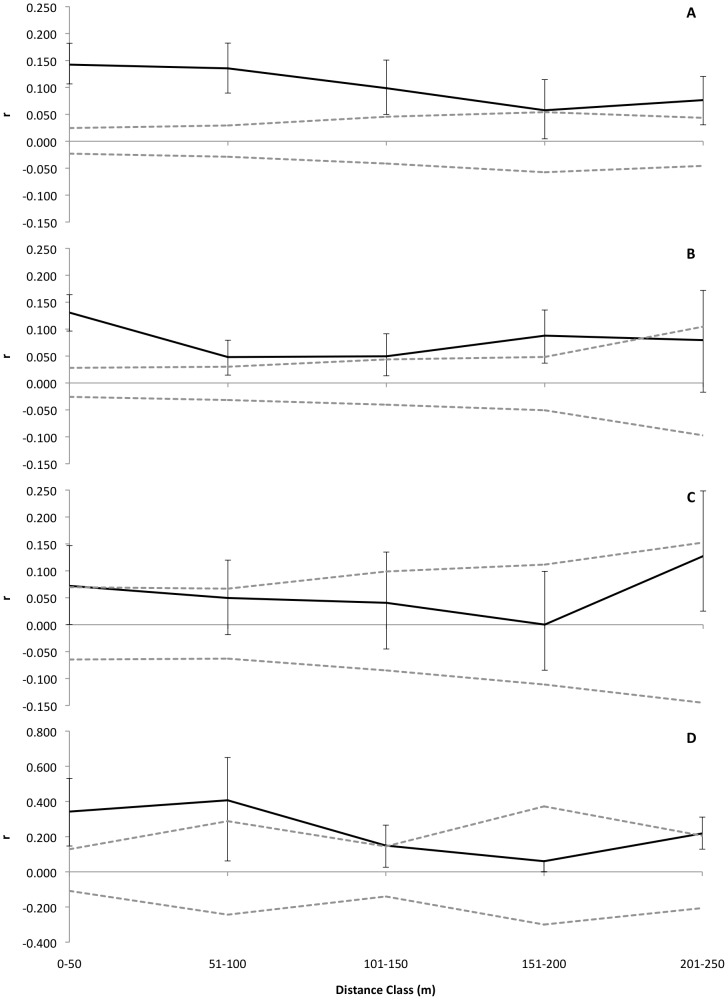
Spatial genetic structure at different life stages of *Protium subserratum*. Correlogram presenting SGS for *P. subserratum* seedlings of different height classes sampled at 14 plots at Ducke: (A) are seedlings up to 20 cm tall; (B) are seedlings from 21 to 40 cm tall, (C) are seedlings from 41 to 100 cm tall and (D) are juveniles and adults taller than 200 cm. The points indicate *r*-values with the error bars and the dashed lines are the upper and lower 95% CI limits around the mean value (*r* = 0) of the null distribution of a random distribution of alleles in space. The number of pairs compared at each distance class is reported in table 3.

**Figure 3:**
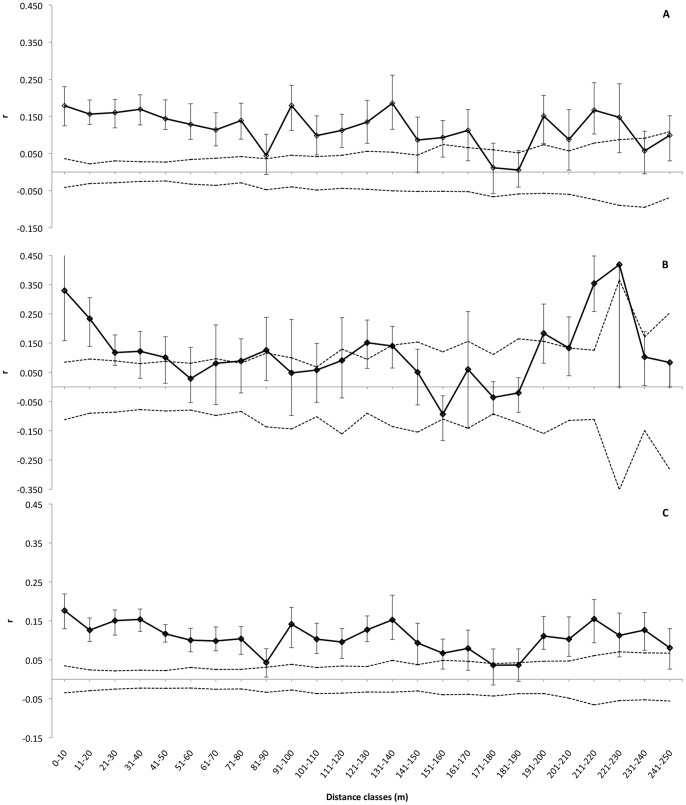
Spatial genetic structure calculated for (A) plants smaller than 40 cm, (B) plants taller than 40 cm and (C) all individuals pooled together. Correlogram of SGS for *P. subserratum* plants sampled at Ducke. The points indicate *r*-values with the error bars and the dashed lines are the upper and lower 95% CI limits around the mean value (*r* = 0) of the null distribution of a random distribution of alleles in space. Distance classes represent 10 m intervals, from 10 to 250 m.

The relatedness of 40 cm seedlings was greater than expected by chance throughout most of the plot, for either 50 m (Fig. 2) or 10 m (Fig. 3) distance interval classes, showing that the results are robust to the choice of distance interval. All four plant size classes had individuals more related to each other than expected by chance in the first distance class (0–50 m – Table 3). The relatedness among individuals decreases either within each plant size class as spatial distance increases (for all plant size classes) and among different plant size classes, as plants grow taller (*r*-values of first-year seedlings>young seedlings>saplings – Fig. 2). Juveniles and adults showed patterns of high relatedness among individuals in the first 100 meters (Fig. 2), although only results from the first distance class (0–50 m) can be considered robust due the small number of pairs compared, especially at the 51–100 m and 201–250 m distance classes. The multiclass test criteria (*ω*) performed separately for each plant size class allows an evaluation of the credibility of autocorrelations, and all four size classes exhibited significant SGS patterns in at least one distance class (Table 3).

**Table 3:** Tests for the significance of autocorrelation at each distance class, presenting number of pairs tested (*n*), autocorrelation *r*-values (*r*) and the multiclass test criteria (*ω*) shown for: i) first-year seedlings (<20 cm); ii) young seedlings (21–40 cm); iii) saplings (41–100 cm); and iv) juveniles and trees (>200 cm).

Distance class (m)		Size class			
		First-year seedlings	Young seedlings	Saplings	Juveniles and trees
0–50	*n*	383	241	41	17
	*r*	0.14***	0.13***	0.07*	0.34***
51–100		257	194	39	3
		0.13***	0.05***	0.05	0.40**
101–150		119	101	20	11
		0.10***	0.05*	0.04	0.15*
151–200		69	77	14	2
		0.06*	0.09***	0.00	0.06
201–250		108	17	8	6
		0.08**	0.08	0.13*	0.29*
Multiclass comparison	*n*	142	130	57	31
	*ω*	61.10***	53.11***	23.70**	41.42***

Significance represented by *<0.05, **<0.01; ***<0.001.

According to the heterogeneity test of relatedness patterns (*r*-values), the three first size classes were not statistically different (Table 4). Only the first-year and young seedlings differed in the second distance class, reflecting a significant drop in the *r*-values of the latter (Fig. 2). First-year seedlings, young seedlings and saplings had relatedness patterns that were different from the juveniles/adult tree class (p = 0.09, p = 0.03 and p = 0.03, respectively).

**Table 4:** Comparisons of autocorrelation patterns of fine-scale genetic structure between size classes of *P. subserratum* at Ducke, Manaus, Brazil.

	*t^2^*					*ω*-test
	Distance classes (m)					
Size class pairs	0–50	51–100	101–150	151–200	201–250	
First-year *vs.* Young	0.17	7.83**	2.05	0.62	0.00	18.61*
First-year *vs.* Sapling	1.47	2.41	0.84	0.76	0.34	12.37
First-year *vs.*Adult/juvenile	4.80*	2.56	0.37	0.00	2.17	16.19^+^
Young *vs.* Sapling	1.01	0.00	0.02	1.83	0.23	6.79
Young *vs.* Adult/juvenile	5.44*	4.27*	1.42	0.03	1.71	19.87*
Sapling *vs.* Adult/juvenile	7.02**	4.15*	1.22	0.12	0.57	20.48*

Statistics and associated probabilities for the differences in SGS between plant size classes, for each distance class (*t^2^*) and for the whole correlogram (*ω*). Significance represented by ^+^<0.1, *<0.05, **<0.01.

We found a strong positive autocorrelation when all sample sizes were pooled across all individuals and plots sampled at Ducke (Fig. 3). This pattern appears to be driven by the high positive autocorrelation among first-year and young seedlings at the plot scale, as the relatedness among juveniles and adults does not present a consistent pattern across the length of the plot.

**Figure 4:**
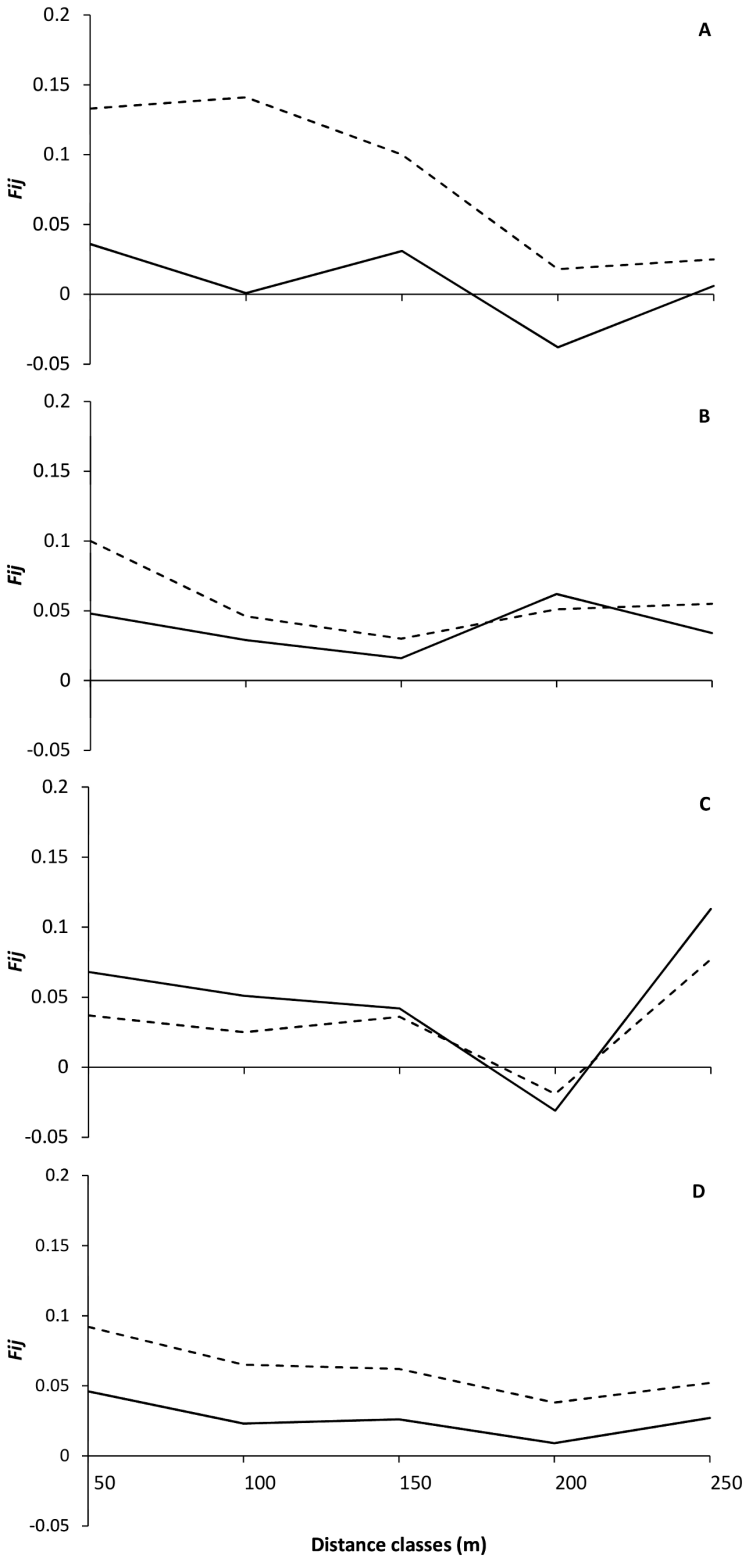
Inter-generational genetic structure analysis: seedling/seedling similarity vs. seedling/adult similarity. Kinship values (*F_ij_*) of adult-seedling pairs (continuous lines) and seedling-seedling pairs (dotted lines) for each plant size class along five distance classes. (A) for first-year seedlings, (B) for young seedlings, (C) for saplings and (D) for all size classes pooled together.

The between-generation SGS analysis, which focused on pairs of individuals that included one adult and one seedling, showed that adults exhibited weak genetic relatedness with nearby seedlings, while within-generation relatedness was generally higher (Fig. 4). This pattern was strongest in the first-year seedlings, which were more genetically similar to other seedlings of the same size class than with nearby adults, especially in the first three distance classes (Fig. 4A). Low relatedness between first-year seedlings and adults indicates that seedlings were dispersed from adults outside the sampling area. Differences in relatedness within and between generations for the young seedling size class decreases as age class increases (Fig. 4B), while the saplings show a shift at the fourth distance class, with individuals from this size class being less related to each other than they are to adults in the plot (Fig. 4C).

### Spatial Genetic Structure and Soil Type

**Figure 5:**
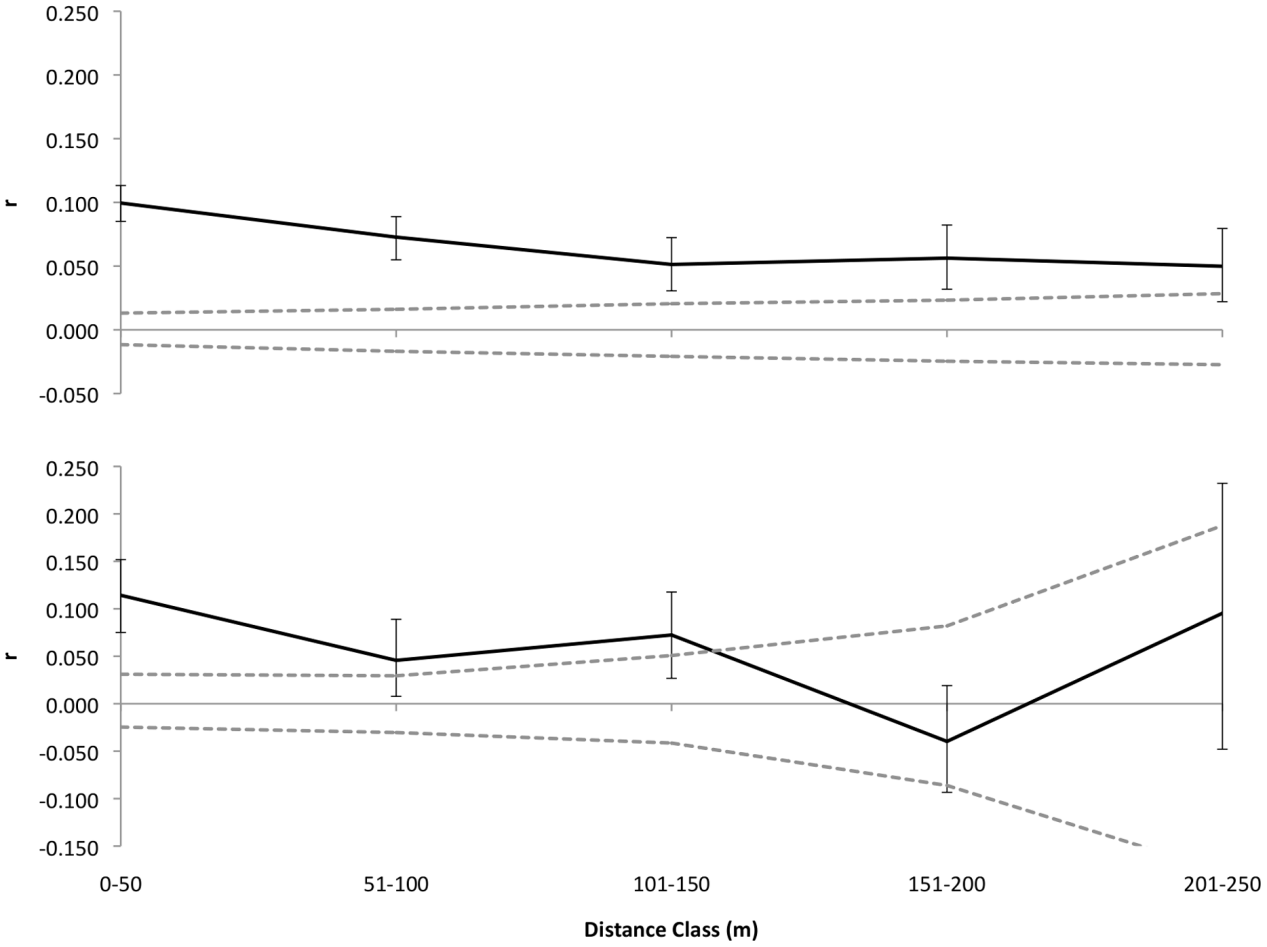
Spatial genetic structure of *Protium subserratum* seedlings at different soil types. Correlograms of SGS for 40 cm height *P. subserratum* seedlings at plots with different soil texture at Ducke: (A) are seedlings in clayey soils and (B) are seedlings in sandy soils. The points indicate *r*-values with error bars and the dashed lines represent 95% CI limits around the mean value (*r* = 0) of the null distribution of a random distribution of alleles in space. The number of comparisons for each distance class is reported in table 5.

The pattern of SGS among first-year and young seedlings was significantly different from random when all samples from all plots were pooled, regardless of their position along the environmental gradient. Since soil texture is a good proxy for topographic position and correlates with plant species composition at our study site [28], [29], we ran two separate SGS analyses based on plot soil texture. The results have shown that soil type influences SGS patterns, as we found positive autocorrelation in the clay plots throughout all distance intervals (Table 5). By contrast, seedlings from sandy soils were significantly positively autocorrelated only in the first three distance classes (up to 150 m – Table 5) after which individuals were no more related with each other than would be expected by chance, since *r*-values fall within the CI area (Fig. 5).

**Table 5:** Tests for the significance of autocorrelation at each distance class for first-year and young seedlings at clay and sandy soils, presenting number of pairs tested (*n*), autocorrelation *r*-values (*r*).

Distance class (m)		Soil type	
		clay	sand
0–50	*n*	1080	369
	*r*	0.10***	0.11***
51–100		632	329
		0.07***	0.05**
101–150		400	149
		0.05***	0.07**
151–200		308	53
		0.06***	–0.04
201–250		206	10
		0.05***	0.09
Multiclass comparison	*n*	196	96
	*ω*	69.08***	43.85***

Multiclass test criteria (*ω*) for testing the hypothesis of “no autocorrelation” at any distance class. Significance represented by **<0.01; ***<0.001.

We found significant differences in SGS for plants inhabiting different soil types (Fisher's *ω* = 18.962, *P = *0.034 – Table 6). This result was due to the low relatedness coefficient (*r*-values lower than expected under the null distribution) of seedlings sampled at the fourth distance class (Fig. 5).

**Table 6:** Comparisons of autocorrelation patterns of fine-scale genetic structure between soil types of *P. subserratum* at Ducke.

Distance class (m)	Sand *vs.* clay plots
0–50	*t^2^ = *0.63
51–100	*t^2^ = *1.73
101–150	*t^2^ = *0.80
151–200	*t^2^ = *7.82**
201–250	*t^2^ = *0.37
Multiclass	*ω* = 18.962*
comparison	

Statistics and associated probabilities for the differences in SGS between seedlings up to 40 cm height located at sandy and clayey soils, for each lag (*t^2^*) and for the whole correlogram (*ω*). Significance represented by *<0.05, **<0.01.

## Discussion

### Patterns across Plant Ontogeny

Spatial genetic structure is often a consequence of limited seed dispersal [44], when seeds and seedlings present higher patterns of relatedness with nearby adults. *Protium subserratum*, despite having dispersers with high mobility potential, has the majority of its fruits deposited beneath the tree crown with extremely high seedling abundances (C.E. Barbosa, pers. observ.). However, when examining the SGS patterns of this tree, we found that younger seedlings presented higher genetic similarity with their neighboring seedlings than with the closest adults. This pattern could result from pulses of seeds coming from distant, unsampled adults further away from the study sites. In addition, the frequency of fruiting cycles might vary among the adults of the same population [8], [17], which could change the SGS observed in a given cohort. Variation in fecundity is another factor that may influence SGS, because adults with greater seed production should be over-represented in the seed shadow composition, yielding more seedlings that are related with each other.

We also found that relatedness decreased between first-year seedlings and saplings. This reduction in relatedness could result from disproportionate mortality near conspecific adults due to density and/or distance-responsive enemies. From a initially highly genetically autocorrelated seed shadow, thinning of closely related individuals occurs as the seedlings grow taller, as shown by the decreasing autocorrelation values from one size class to the next. One mechanism that would cause this pattern is if anti-herbivore defenses were variable within populations and closely related plants express very similar anti-herbivore defenses [12], [13]. Specialist natural enemies may use these defenses as cues, and genetically distant individual seedlings may escape notice when in a group of conspecifics. Strong evidence of higher mortality within closely related seedlings was described recently in Choo et al. [45], in which the proportion of seedlings dispersed near the maternal parent decreased threefold throughout the development stages evaluated, while the proportion of non-related seedlings remained similar. Alternatively, fungal pathogens may have strong negative effects on young seedlings near parent trees [6], but both herbivore and pathogen negative effects may subside as seedlings mature.

The differences we found in relatedness between first-year and young seedlings agrees with other studies that have evaluated SGS across different life stages [46], [47]. Zhou & Chen [2] detected SGS dissipation as seeds and seedlings developed to the sapling and adult stages, but they could not determine the stage at which density dependent mortality began operating because they did not measure young seedlings. Hamrick et al. [48] also found SGS at seedling and sapling stages and Hardesty et al. [49] identified differences on SGS patterns when comparing size classes of a tropical tree species, but unlike our study they did not detect SGS among individuals at the adult stage.

Seeds from distant parents dispersed from outside the study area might accumulate over multiple fruiting years, causing the decrease of relatedness as seedlings age. In this scenario we would expect the youngest seedlings to be closely-related to local adults, but that genetic-relatedness would be reduced as seedlings grew older [40]. However the between-generation analysis (Fig. 4) does not support this hypothesis as the observed youngest seedlings were less related to the adults than to each other. In fact relatedness was more and more similar to adults as the cohorts aged, which might help to explain the high relationship values among juvenile and adult tree individuals in the first distance classes. The adults and juveniles were more related to each other than would be expected by chance, which is contrary to the tendency for relatedness to decrease, which is what we found for earlier stages of development. The adults of the first distance class had the highest relatedness value (n = 17, *r* = 0.34, more than twice the first-year seedlings’ *r*-value). However, the *r*-values among juveniles and trees, as well as the *r*-values for the greater distance classes of the saplings, should be interpreted cautiously as they are derived from a small number of comparisons. Nevertheless, the pattern of greater relatedness within adult and juvenile trees is strengthened by the fact that the within-generation relatedness values that we observed were much higher than the between-generation relatedness for the first size class, and these values showed a clear tendency to decrease as seedlings aged, until the sapling stage where the plants’ relatedness values are more similar to the adults.

Our finding of the reduction in relatedness within seedlings as plants grow older, yet exhibiting higher relatedness values with adults, could be explained by a life-stage conflict, as predicted by Schupp [50], in which the trade-off between the positive and negative aspects of being nearby a reproductive adult should vary according to the ontogenetic stage. Thus, being close to an adult might be much more detrimental to young seedlings than for saplings and adults, as the higher probability of herbivore and/or pathogens attack greatly surpasses the advantage of being in the same site of a reproductive adult (i.e., an “optimal” site for establishment) in an early life stage. Additionally, contrary to the prediction of Janzen’s graphical model of high seedling mortality at specific distances from adults [9], [11] and supporting studies such as Alvarez-Loayza & Terborgh [20] that observed extremely high seedling mortality, many *P. subserratum* seedlings in Ducke do escape mortality in close proximity to adults. The clustering of genetically closely related juveniles and adults might result from uneven seed distribution, with seeds from the same maternal tree being dispersed to the same place [51], [52], resulting in clumping of related individuals. Besides these possibilities, we would also expect that most seedlings would derive from nearby individuals of *P.* *subserratum*, considering the deposition of large numbers of seeds near the maternal adult year after year compared to a minor contribution of seeds dispersing from adults at greater distances. In that context, the probability that the seedlings that do escape from predation will be related to the nearest adults is higher. Our results stand in contrast to studies that found individuals related to each other, but occurring far away from the maternal individual [53]–[55]. Finally, closely related adults may themselves relate to the reproductive success of a few previous adults from past generations, and may thus be a consequence of episodic reproduction or founder effects.

Steinitz et al. [56] found results contrasting with our study in a temperate forest in a study of older pine saplings and adult trees. They reported that density dependent mortality strengthened with age, working as a spacing mechanism all the way to adulthood. The authors argue that a possible explanation for this pattern would be the increase in competition with conspecific adult trees. Our results are more in accordance with those found by Latouche-Hallé et al. [57] for a tropical tree species at French Guiana, where genetic relatedness was higher in adults that were close together, which they credited to overlapping generations in adult cohorts being more dissimilar at greater distances.

### Patterns across Soil Types

Even within the same population of *P. subserratum*, and along a relatively short environmental gradient, relatedness patterns appear to be significantly different between soil types. In slopes and ridges, where the clay plots were located, the relatedness values among individuals 150 m distant from each other were higher than in the lower areas (the sandy plots of the valleys), in which relatedness between individuals was no different than what would be expected by chance. This pattern may be explained by two interconnected factors: soil type and topography.

Soils in central Amazonia are notoriously poor and sandy soils are significantly less fertile than clay soils. Differences in seed output caused by soil nutrient properties could change the size of seed shadows, thus affecting SGS patterns. But since tree diameter is a good predictor of fecundity potential [58], [59], and at Ducke the size of *P. subserratum* trees is similar across the soil texture gradient (r^2^ = –0.03, p = 0.72, unpublished data), it is reasonable to expect similar seed output throughout the soil texture gradient. Variation in the number of reproductive adults may also influence SGS as a result of overlapping seed shadows [48], but the abundance of *P.* *subserratum* adults is also similar along the soil texture gradient, therefore the chances of overlapping seed shadows should be constant throughout the gradient.

Subtle environmental gradients not only may affect an individual’s growth [60], but they can also influence the chemical defenses against predation [13] and ultimately act as a selective agent on genotypes. Despite the evidence of stronger herbivore pressure in a forest on clay soil sites [21], the impact of leaf attack in poor soils is higher, because the replacement of the leaf tissue lost to herbivores should be much more difficult in a low nutrient environment [21]. Stronger herbivore impacts on mortality in sandy habitats could lead to higher density/distance related mortality and ultimately result in the lower genetic autocorrelation among seedlings in sandy soils.

Nevertheless, density/distance dependent mortality might be overwhelmed by abiotic factors that regulate establishment success. We found higher mortality of *P. subserratum* seedlings in clay plots (45%) than in sandy plots (16%) in a parallel study conducted during a two year period (C. E. Barbosa, unpublished data), which is the opposite of what would be expected because clay plots exhibited stronger patterns of spatial genetic structure than did sandy plots. The increased mortality might be related to drought during the dry season, which tends to have stronger effects in clay soil areas that are located away from streams. However drought-related mortality probably happens regardless of the proximity to conspecific adults and should not influence the SGS patterns found here.

Additionally, valleys may receive a more genetically diverse seed influx from higher places, due a topographic effect of seeds being carried down by gravity as well as by rainwater from the steeper areas. Even in the absence of rain, seeds from trees in higher areas have a higher probability of being dispersed to the valleys by birds, gravity or wind than seeds from valley trees have of being moved to higher sites. This could lead to a greater genetic diversity in valleys, and hence to a lower genetic relatedness among seedlings.

Edaphic gradients and topography have a large influence in driving plant species composition in the Amazon [29], [61] and density/distance-dependent mortality could be one of the mechanisms involved. The evidence we found for contrasting JC effects between sandy and clay soils at Ducke may be part of a more general mechanism influencing community-level patterns in different soil types and could contribute to the high turnover of plant community composition in the Central Amazonian lowlands [30].

### Conclusions

We observed spatial genetic structure patterns in all evaluated stages of *P. subserratum*, with a tendency for relatedness to decrease across the early stages of development. However, this tendency was not found in the adults, which presented high SGS. That may be an indication that particular genotypes are better adapted to a very local scale, resulting in more genetically spatially-autocorrelated adults, as they would be more likely to share traits for the same abiotic and biotic environments. The different SGS patterns that we found between clayey and sandy soils may represent another layer of trait selection acting on a higher spatial scale.

The presence of SGS across environments in seedlings of a species so broadly distributed across Amazonia could be an indication that *P. subserratum* may be more locally adapted to soil type than previously assumed, and shows the impact that habitat heterogeneity can have on genotype distribution. Future studies should investigate if the SGS differences among environments are sustained across subsequent plant development stages including among adults in different soil types. Environmental heterogeneity across the landscape is known to be a driver of speciation [62] with the action of ecological selection on genotypes. Fine et al. [63], when analyzing extremely poor soils (white sands) in contrast with more fertile clay soils, found that soil type interacts with herbivore pressure leading to habitat specialization. We speculate that our findings here might represent an intermediate stage in that process, as the downslope sandy soils at Central Amazon are not as poor as white sand soils, therefore should represent a less extreme gradient. Nevertheless the differences between habitats were sufficient to establish diverse patterns of genetic structure among environments.

### Data Accessibility

Sampling locations, soil type, tree sizes and microsatellite genotypes for all individuals are available at the data repository of the Brazilian Program for Biodiversity Research (PPBio) at http://peld.inpa.gov.br/knb/metacat/prisouza.29.13/peld.
